# Otosyphilis: A rare case of reversible hearing loss in a young man with secondary syphilis

**DOI:** 10.1016/j.idcr.2022.e01666

**Published:** 2022-12-20

**Authors:** Enas W. Sarsak, Walid E. Omer, Ahmad A. Al Bishawi, Muna Al Maslamani, Alaaeldin Abdulmajed Basher Abdulmajed

**Affiliations:** a Department of Medicine, Hamad Medical Corporation, Doha, Qatar; b Department of ENT, Hamad Medical Corporation, Doha, Qatar

**Keywords:** Otosyphilis, Neurosyphilis, Hearing loss, Sexually transmitted diseases, Syphilis

## Abstract

**Background:**

Over the last decade, it has been noticed a significant increase in the number of cases of syphilis with a concurrent increased number of patients presenting with syphilis-related complications. Otosyphilis is a well-known complication of syphilis that most of the time, can lead to irreversible hearing loss, especially with delayed diagnosis and treatment. A high index of suspicion is needed for an accurate diagnosis of otosyphilis. Complete audiologic recovery is rare but still possible with the appropriate treatment.

**Case report:**

Herein, we describe a case of reversible hearing loss secondary to otosyphilis in a young healthy man who was initially diagnosed and treated as a case of secondary syphilis, and presented later to the clinic with unilateral tinnitus and hearing loss. Audiology findings were consistent with asymmetric sensorineural hearing loss. Fortunately, complete recovery of hearing was achieved after treatment with a 14-day course of intravenous penicillin.

**Conclusion:**

Otosyphilis is one of the rare presentations of syphilis; thus, the diagnosis is often missed or delayed. Prompt diagnosis and treatment can help prevent the occurrence of permanent hearing loss.

## Background

The number of cases of syphilis are increasing worldwide among men and women, with almost all new syphilis infections being sexually acquired. Syphilis, often called the great mimicker, can present with multisystem involvement. One of its rare presentations is Otosyphilis, an important complication that can lead to the devastating outcome of permeant hearing loss if left untreated.

## Case report

A 25-year-old Qatari male with no medical or surgical history presented to the Infectious Diseases clinic with a history of skin rash for one month. The skin rash was diffuse, involving the trunk, extremities, palms, and soles. It was light brown in color and non-itchy, associated with malaise and myalgia but with no associated fever or sore throat.

Two weeks before the onset of the skin rash, the patient noticed a non-painful ulcer over his penile shaft. No purulent discharge was seen, and the ulcer healed spontaneously within four weeks.

The patient denied any associated headache, neck stiffness, nausea, vomiting, blurred vision, or hearing loss, and no associated urethral discharge, urinary, gastroenterology, or musculoskeletal symptoms. Two months earlier, the patient had gone on vacation abroad and had multiple unprotected sexual encounters with sex workers and women. His initial assessment at the clinic was normal except for a scaly brownish macular-papular skin rash over the trunk and extremities, including palms and soles. Otherwise, his clinical examination, including a thorough neurological examination, was normal.

Based on this typical skin rash, a diagnosis of secondary syphilis was suspected, and serum treponemal as well as non-treponemal tests were ordered, which came back positive with RPR titer 1:32. Subsequently, complete sexually transmitted infection (STI) screening tests were ordered, including HIV-1/2 Ag/Ab Combo test, and all returned negative.

The patient was initially treated for secondary syphilis with one dose of intramuscular Benzathine penicillin 2.4 million units. However, one week later, the patient presented to the clinic complaining of a new onset left ear tinnitus and fullness but denied hearing loss.

**Fig. 1:**
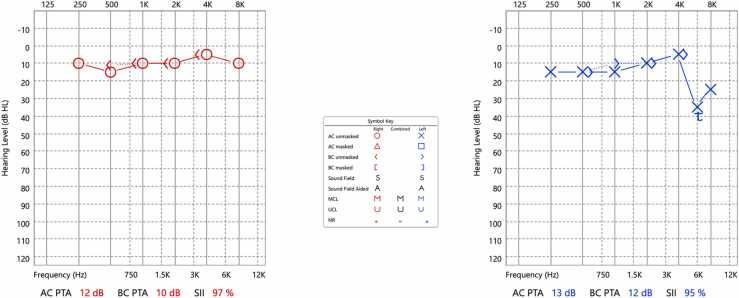
Pure Tone Audiogram (Initial) revealed hearing within normal limits bilaterally apart from an asymmetrical left sensorineural dip at 6 kHz of 35 dB.

The patient was admitted to the communicable disease center (CDC) as a case of suspected neurosyphilis/otosyphilis for further evaluation, where ENT assessment was arranged immediately. Pure Tone Audiometry confirmed an asymmetrical left sensorineural hearing loss with a dip at 6 kHz of 35 dB on the left side (Fig. 1). His tympanometry revealed Type "A" bilaterally, showing normal tympanic membrane mobility bilaterally, as ear canal volume, peak pressure, and static compliance were within normal limits. Computed Tomography (CT) scan of the temporal bone was unremarkable, with no evidence of inflammation. However, a lumbar puncture was not performed as the patient declined the procedure.

**Fig. 2:**
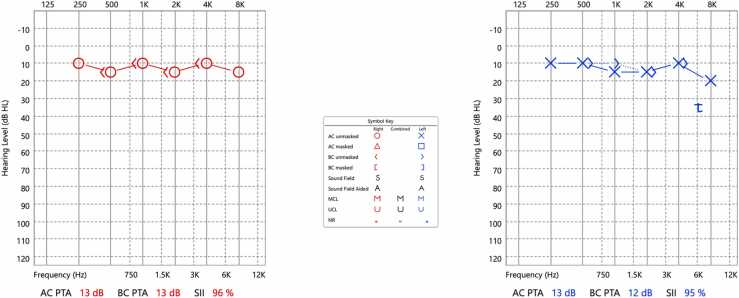
Pure Tone Audiogram (post treatment) revealed hearing within normal limits bilaterally with resolution of the initial left sensorineural dip at 6 kHz.

The patient was started on IV penicillin G (3 million Q 4 hrs.), and he completed a total of 14 days duration with no complications. A follow-up audiogram was done one month later (Fig. 2) and was within normal limits. A follow-up serum RPR showed a significant titer reduction (repeat RPR 1:4).

## Discussion

Syphilis is a sexually transmitted disease caused by the spirochete bacteria Treponema pallidum subspecies pallidum (T. pallidum). Despite the current advances in this disease diagnosis, control and treatment, its recognition is still not well-understood or often not considered by many physicians as it is so-called the "great mimicker" due to the various clinical manifestations it produces, which often overlap with many other medical conditions making it overlooked; additionally, its diagnosis could be even heralded by a period of latency and active disease[1]. This spirochete (Treponema) can't be cultivated in vitro, and the diagnosis usually relies on serology testing and identifying its distinct spiral movements on darkfield microscopy. It can't survive outside the human host, and its primary way of transmission is either sexually or vertically during pregnancy [2]. Without adequate treatment, the disease can advance further into multiple clinical stages, which is classified as early syphilis "primary, secondary and early latent syphilis" (less than 12 months) - which is contagious, and as late syphilis "late latent and tertiary syphilis" (more than 12 months) which is rarely infectious but can cause cardiovascular or irreversible neurological complications after several years [3]. After inoculation, early in the disease, T. pallidum can disseminate throughout the body, including to the central nervous system (CNS), referred to as neurosyphilis, which can occur at any syphilitic stages, i.e., primary, secondary, and tertiary [4]. However, it's still not well understood why neurosyphilis complications can affect some hosts, but the host and bacterial factors likely play a role [5].

The term otosyphilis is considered a manifestation of neurosyphilis spectra, which can develop in congenital or acquired syphilis. The pathophysiology behind this manifestation could be attributed to the involvement of the 8th cranial nerve within or outside of the CSF space, the cochleovestibular apparatus, or the temporal bone by T. pallidum [6], where early in the disease dissemination, spirochetes might directly invade the perilymph of the inner ear, causing inflammation confined to the bony labyrinth [7]. Additionally, it has been noted that osteitis and periostitis of the temporal bone and involvement of the ossicles in the middle ear in patients with a long-standing infection or a history of untreated congenital syphilis could play a role in this manifestation [8].

The diagnosis of Otosyphilis could be challenging since the clinical manifestation may mimic other etiologies of hearing loss. Hence, diagnosis is usually based on audiometry abnormalities in patients with current or recent syphilis "suggested by clinical manifestation and supported by treponemal and nontreponemal serologic testing in evaluating past nontreponemal serologic titers in those with a history of treated disease" and exclusion of other diagnoses [9]. Luckily in our patient's scenario, he presented shortly after receiving the diagnosis of secondary syphilis with hearing symptoms, which alerted us immediately to consider this complication in our working diagnosis.

Reviewing the literature, in a retrospective study evaluating Otosyphilis of 85 cases, the most common presenting symptoms were hearing loss (90.6%), tinnitus (72.9%), and vertigo (52.9%) [10]. The cerebrospinal fluid analysis was positive for VRDL in 5.4%, which further emphasizes that common hearing loss in adults is sensorineural. However, conductive hearing loss may be apparent in those with longstanding, unilateral, or bilateral disease, which is often sudden in onset and rapid progression [11]. Reported symptoms and audiometry findings might differ as some patients may report unilateral hearing impairment subjectively but are found to have a bilateral congenital form [12]. It has been suggested that up to about 17% of individuals with syphilis can have sensorineural hearing loss at a certain point in their disease course [13].

The positivity of CSF VDRL could be supportive to an established diagnosis of neurosyphilis and is still recommended by CDC for all suspected otosyphilis cases [9]; however, it is not always required to make a diagnosis of Otosyphilis given the low sensitivity of the test [14]. Our patient declined the procedure. Nevertheless, he showed excellent response to treatment clinically, with subsequent audiometric testing and serologically.

The suggested treatment regimen usually includes intravenous penicillin G 10–14 days, and sometimes adjunctive steroid is used to reduce meningeal inflammation with better outcomes, and reduced risk of Jarisch-Herxheimer reaction, with success rates ranging from 15% to 18% in congenital syphilis [15]. On the other hand, in adulthood acquired form, there has yet to be a clear consensus regarding this regimen's success rate. Some experts suggested that penicillin treatment is mainly targeted to stop disease progression instead of curing it by completely restoring hearing ability. However, as a consensus, this disease can be easily treated if caught early. Otherwise, arrest of progression seems to be the only option [16], [17]. Other suggested antimicrobial options include Ceftriaxone (IM or IV) [18] and oral Doxycycline 400 mg/day for 21 days [19], with limited data about its efficacy. Post-treatment follow-up, including clinical assessment, audiometric improvement, and serological response by serofast state, defined as a 4-fold drop in nontreponemal serologic titers up to 12 months after treatment for primary, secondary, or early latent syphilis and 24 months for late latent disease [20], which all seen fortunately in our patient during his follow up.

## Conclusion

In the context of rising syphilis rates worldwide, a rise in neuro-, oto- and ocular cases of syphilis can be anticipated. All patients should be carefully screened with a complete history and neurological examination regardless of the syphilis stage. While otosyphilis is one of the few forms of the potentially curable SNHL, diagnosing otosyphilis is often challenging, requiring a high index of clinical suspicion. Otherwise, it can be easily missed leading to irreversible hearing loss.

## CRediT authorship contribution statement

**Enas W. Sarsak:** manuscript writing, literature review, study design. **Walid E. Omer:** Writing, data collection. **Ahmad A. Al Bishawi:** Writing – review & editing. **Muna Al Maslamani:** Study design. **Abdulmajed Basher Abdulmajed:** Writing – review & editing, study design.

## Ethical approval

The institutional review board has approved this study under the number “MRC-04-22-298”.

## Consent

Informed consent for case report has been waived by the institutional review board (IRB).

## Conflicts of interest

No conflicts of interest to declare.
